# Supplementary material for: Dual RNA Sequencing of Mycobacterium tuberculosis-Infected Human Splenic Macrophages Reveals a Strain-Dependent Host–Pathogen Response to Infection
Source: Int J Mol Sci. 2022 Feb 4;23(3):1803. doi: 10.3390/ijms23031803 (PMC8836425; doi:10.3390/ijms23031803)
Supplement: Supplementary file 1 [file ijms-23-01803-s001.zip › ijms-1491819-supplementary/ijms-1491819-Supplementary text-final.pdf]

International Journal of *Molecular Sciences*

MDPI

Article

Dual RNA Sequencing of *Mycobacterium tuberculosis*-Infected Human Splenic Macrophages Reveals a Strain-Dependent Host–Pathogen Response to Infection

Víctor A. López-Agudelo [1,2,†], Andres Baena [1,†], Vianey Barrera [3], Felipe Cabarcas [4], Juan F. Alzate [5], Dany JV Beste [6], Rigoberto Ríos-Estepa [2] and Luis F. Barrera [1,*]

[1] Grupo de Inmunología Celular e Inmunogenética (GICIG), Instituto de Investigaciones Médicas, Facultad de Medicina, Universidad de Antioquia Medellín, 050010, Colombia; v.lopez-agudelo@ikmb.uni-kiel.de (V.A.L.-A.); andres.baenag@udea.edu.co (A.B.)

[2] Grupo de Bioprocesos, Facultad de Ingeniería, Universidad de Antioquia, Medellín, 050010, Colombia; Rigoberto.rios@udea.edu.co

[3] Programa de Ingeniería Biológica, Universidad Nacional de Colombia, Sede Medellín, Medellín, 050010, Colombia; vpbarrerae@unal.edu

[4] Grupo Sistemas Embebidos e Inteligencia Computacional (SISTEMIC), Facultad de Ingeniería, Universidad de Antioquia Medellín, 050010, Colombia; felipe.cabarcas@udea.edu.co

[5] Centro Nacional de Secuenciación Genómica (CNSG), Sede de Investigación Universitaria (SIU), Facultad de Medicina, Universidad de Antioquia Medellín, 050010, Colombia Colombia; jfernando.alzate@udea.edu.co

[6] Department of Microbial Sciences, Faculty of Health and Medical Science, University of Surrey, Guildford, GU2 7XHUK; d.beste@surrey.ac.uk

* Correspondence: luis.barrera@udea.edu.co

† These authors contributed equally to the study.

Citation: López-Agudelo V.A.; Baena, A.; Barrera, V.; Cabarcas, F.; Alzate, J.F.; Beste, D.JV.; Ríos-Estepa, R.; Barrera, L.F. Dual RNA Sequencing of Mycobacterium tuberculosis-Infected Human Splenic Macrophages Reveals Strain-Dependent Host-Pathogen Response to Infection. *Int. J. Mol. Sci.* **2022**, *23*, 1803. https://doi.org/10.3390/10.3390/ijms23031803

Academic Editor: Alexander S. Apt

Received: 17 November 2021
Accepted: 28 December 2021
Published: 4 February 2022

Publisher's Note: MDPI stays neutral with regard to jurisdictional claims in published maps and institutional affiliations.

Copyright: © 2021 by the authors. Licensee MDPI, Basel, Switzerland. This article is an open access article distributed under the terms and conditions of the Creative Commons Attribution (CC BY) license (http://creativecommons.org/licenses/by/4.0/).

Supplementary

Host-Mtb genome-scale model building and interdependence test

The following steps were used for merging the sMtb2.0 model with the host-pathogen model of Zimmerman and colleagues [30]: (i) Stoichiometric matrix merge, (ii) gene nomenclature update, (iii) interdependence test and (iv) validation of physiological capabilities.

To merge the stoichiometric matrices of sMtb2.0 and the macrophage model, sixty-one transport reactions (amino acids, fatty acids, nucleotides, metal ions, sugars, glycerol, and others) were used to share metabolites between the macrophage network and sMtb2.0 through a common phagosome compartment.

Tags such as "ma" and "mt" were used to differentiate reactions belonging to macrophage and Mtb, respectively. Moreover, we updated the host-pathogen gene-protein-reaction rules by mapping the reactions of the macrophage metabolic network with the human metabolic network Recon2.2 [105] that allowed changing the old NCBI identifiers with the HGNC identifiers.

The original biomass objective function of sMtb2.0 was modified to better represent the physiological conditions of Mtb in the intracellular environment. Bordbar and Colleagues revised the biomass objective function of the iNJ661 Mtb model and modified it so as to fit gene expression data derived from *in vivo* and *in vitro* infection [106]. Relevant modifications to iNJ661 objective function included an increase in coefficients for amino acids and mycobactin, and a decrease in coefficients for phospholipids and ATP maintenance. As iNj661 and sMtb2.0 are metabolic networks for the same organism (Mtb H37Rv), we decided to make the use modifications for the *in vitro* biomass of sMtb2.0 and therefore, to obtain a biomass pseudo-reaction for the intracellular environment (Table S8).

We evaluated the consistency of the coupled metabolic networks by computing a Flux variability analysis (FVA) [109] with open macrophage uptake rates and maximizing

the Mtb biomass pseudo-reaction (Table S9). The FVA showed that from 61 metabolites transported to macrophage phagosome, only six could not be taken by Mtb (blocked reactions); they were Cytidine, Ethanolamine, Glycoaldehyde, H_2CO_3, Maltose, and Trehalose. For the first four, the sMtb2.0 model has not transport reactions, although sMtb2.0 can properly take up CO_2 from the phagosome. For the last two, Maltose and Trehalose, the macrophage network contains enzymes that extracellularly hydrolase both molecules, impeding them to be transported to the phagosome, and therefore to sMtb2.0:

$$h2o[e] + \ malt[e] \rightarrow \ 2\ glc - D[e]\ (\mathrm{alpha - glucosidase, extracellular}, HGNC\text{: }7043) \tag{1}$$

$$h2o[e] + \ tre[e] -> \ 2\ glc - D[e](alpha, alpha - trehalase, HGNC\text{: }12266) \tag{2}$$

The merged model contains 4773 reactions, 3631 metabolites, 2467 genes, and eight compartments (cytoplasm [c], endoplasmic reticulum [r], Golgi apparatus [g], lysosome [l], mitochondria [m], nucleus [n], phagosome [ph], and extracellular environment [e]).

An interdependence test was developed for checking the effective metabolic interaction between pathogen and host models as recommended by Jamshidi and Raghunathan (5) (see Equations 3-8).

The interdependence test is usually tested on the biomass pseudo-reaction of both host and pathogen networks. The interdependence test is performed in two steps:

I. Compute the optimal host biomass, $v_{hp}^{\mathrm{BM,h}}$,in the host-pathogen model, then the lower bound of the host biomass reaction is fixed to a specified value $(1 - \varepsilon_1)$, followed by optimization of the pathogen biomass:

$$\mathrm{For}\ \alpha_1 = \max(v_{hp}^{\mathrm{BM,h}}) \tag{3}$$

$$\text{set: lower bound}\ \left(v_{hp}^{\mathrm{BM,h}}\right) \geq (1 - \varepsilon_1)\alpha_1 \tag{4}$$

$$\max(v_{hp}^{\mathrm{BM,p}}) = \beta_2 \tag{5}$$

II. Compute the optimal pathogen biomass production, $v_{hp}^{\mathrm{BM,p}}$, in the host–pathogen model, fix the lower bound of the pathogen biomass reaction to a specified value $(1 - \varepsilon_2)$ and then optimize the biomass of the host:

$$\mathrm{For}\ \beta_1 = \max(v_{hp}^{\mathrm{BM,p}}) \tag{6}$$

$$\text{set: lower bound}\ \left(v_{hp}^{\mathrm{BM,p}}\right) \geq (1 - \varepsilon_2)\beta_1 \tag{7}$$

$$\max(v_{hp}^{\mathrm{BM,h}}) = \alpha_2 \tag{8}$$

If $\alpha_1 \neq \alpha_2$ and $\beta_1 \neq \beta_2$ there is metabolic interdependence and coupling between the two metabolic network models.

We would expect that the metabolism of Mtb is affected by the macrophage metabolism and vice versa. To carry out this test, we chose the macrophage and mycobacteria biomass functions as objective functions. These functions connect several different pathways within each organism and therefore will likely affect the global host-pathogen interaction [104]. Likewise, as recommended by Jamshidi and Raghunathan [104], we chose an empirical coefficient $\varepsilon = 0.01$. After setting the medium constraints (RPMI medium), we ran the optimization problem for each step obtaining $\alpha_1 = 0.0486$, $\beta_2 = 2.2543e - 04$, $\beta_1 = 0.0127$, and $\alpha_2 = 0.0152$. Hence, as $\alpha_1 \neq \alpha_2$ and $\beta_1 \neq \beta_2$,we conclude that there is a metabolic interdependence between both pathogen and host metabolic network models

and each perturbation on the macrophage metabolic network will have consequences for the sMtb2.0 metabolic network and vice versa.

Table S1. Host–pathogen metabolic network features*.

Host-Pathogen Network attributes	Value
Reactions	4773
Metabolites	3631
Genes	2467
Compartments	8
**Interdependence**	**Value**
α_1- Macrophage biomass maintenance - 1 step	0.0486
β_2 - Maximal biomass Mtb – 1 step	0.0002543
β_1 - Maximal biomass Mtb – 2 steps	0.0127
α_2 - Macrophage biomass maintenance – 2 steps	0.0152

***This table contains the global attributes of the host–pathogen network, the results of the interdependence test were obtained with a traditional flux balance analysis simulation.**

References

30.Zimmermann, M.; Kogadeeva, M.; Gengenbacher, M.; McEwen, G.; Mollenkopf, H.-J.; Zamboni, N.; Kaufmann, S.H.E.; Sauer, U. Integration of Metabolomics and Transcriptomics Reveals a Complex Diet of Mycobacterium tuberculosis during Early Macrophage Infection. *mSystems* **2017**, *2*, e00057-17, https://doi.org/10.1128/msystems.00057-17.

112.Jamshidi, N.; Raghunathan, A., Cell scale host-pathogen modeling: Another branch in the evolution of constraint-based methods. *Front Microbiol.* **2015**, *6*, 1032, https://doi.org/10.3389/fmicb.2015.01032.

113.Swainston, N.; Smallbone, K.; Hefzi, H.; Dobson, P.D.; Brewer, J.; Hanscho, M.; Zielinski, D.C.; Ang, K.S.; Gardiner, N.; Gutierrez, J.M.; et al. Recon 2.2: from reconstruction to model of human metabolism. *Metabolomics* **2016**, *12*, 1–7, https://doi.org/10.1007/s11306-016-1051-4.

114.Bordbar, A.; Monk, J.M.; King, Z.; Palsson, B.O. Constraint-based models predict metabolic and associated cellular functions. *Nat. Rev. Genet.* **2014**, *15*, 107–120, https://doi.org/10.1038/nrg3643117. Mahadevan R, Schilling C. The effects of alternate optimal solutions in constraint-based genome-scale metabolic models. Metabolic engineering. 2003;5:264–76. .

117.Mahadevan, R.; Schilling, C.H. The effects of alternate optimal solutions in constraint-based genome-scale metabolic models. *Metab. Eng.* **2003**, *5*, 264–276, https://doi.org/10.1016/j.ymben.2003.09.002.
