# Supplementary material for: Dual RNA Sequencing of Mycobacterium tuberculosis-Infected Human Splenic Macrophages Reveals a Strain-Dependent Host–Pathogen Response to Infection
Source: Int J Mol Sci. 2022 Feb 4;23(3):1803. doi: 10.3390/ijms23031803 (PMC8836425; doi:10.3390/ijms23031803)
Supplement: Supplementary file 1 [file ijms-23-01803-s001.zip › ijms-1491819-supplementary/Supplementary Figures v2.pdf]

Supplementary Figures

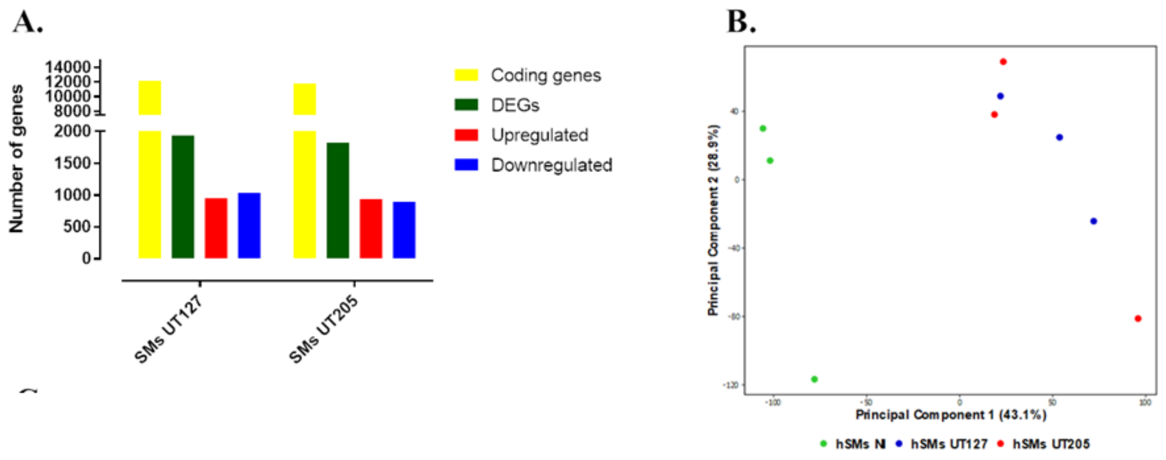

Figure S1. ***Distribution of coding, differentially expressed, upregulated, and downregulated genes in hSMs infected with the Mtb strains UT127 and UT205.*** (**A**) hSMs infected with Mtb UT127 expressed 12,179 protein-coding genes from which 1,986 (16.3%) were differentially expressed compared to non-infected cells. 951 (47.9%) were upregulated, and 1,035 (52.1%) were downregulated. In comparison, 11,840 protein-coding genes were expressed by hSMs infected with UT205, from which 1,825 (15.4%) were differentially expressed compared to non-infected cells. 935 (51.2%) were upregulated, and 890 (48.2%) were downregulated. (**B**) sample distribution based on the Principal Component Analysis of non-infected and Mtb-infected samples. (**C)** As determined by PCA, the distribution of intracellular biological replicas of UT127 (1-3) and UT205 (1-3) and axenic cultures of UT127 and UT205 in Sauton's media. SM1-3, Non-infected macrophages; MOUT127, macrophages infected with UT127; MOUT205, macrophages infected with UT205; UT127 1-3, UT205 1-3, clustering of Mtb transcriptomes in infected macrophages; UT127- and UT205-Sauton, clustering of Mtb UT127 and UT205 in Sauton's axenic medium.

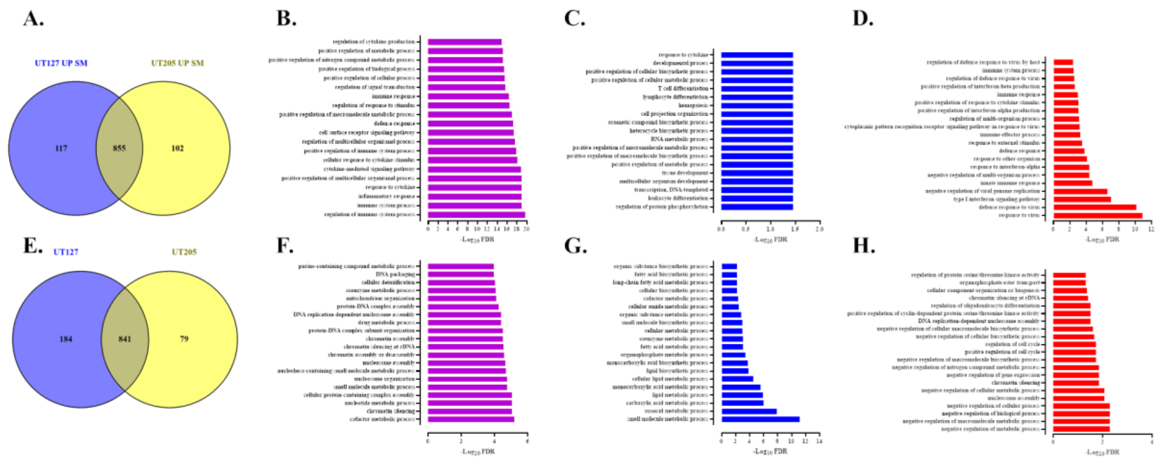

Figure S2. ***Biological processes associated with common upregulated and downregulated human splenic macrophages in vitro infected with Mtb UT127 or UT205***. Human splenic macrophages (hSMs) were infected for 6 hours with a multiplicity of infection (MOI) of 10:1. (**A,E)** Venn diagram indicating the amount of significantly upregulated (**A**) or downregulated (**E**) genes (Log_2F=1.5; p<0.05; FDR<0.05). (**B,F**) display the top 20 common biological processes (BP) associated with the upregulated (**B**) or downregulated (**F**) genes. (**C,G**) display the top 20 unique biological processes associated with the upregulated (**C**) and downregulated (**G**) genes expressed by hSMs infected with Mtb UT127. (**D**,**H**) display the top 20 unique biological processes associated with the upregulated (**D**) and downregulated (**H**) genes expressed by hSMs infected with Mtb UT205. The list and significance of the biological processes were obtained after analyzing the String database has significantly expressed (up-or downregulated) genes.

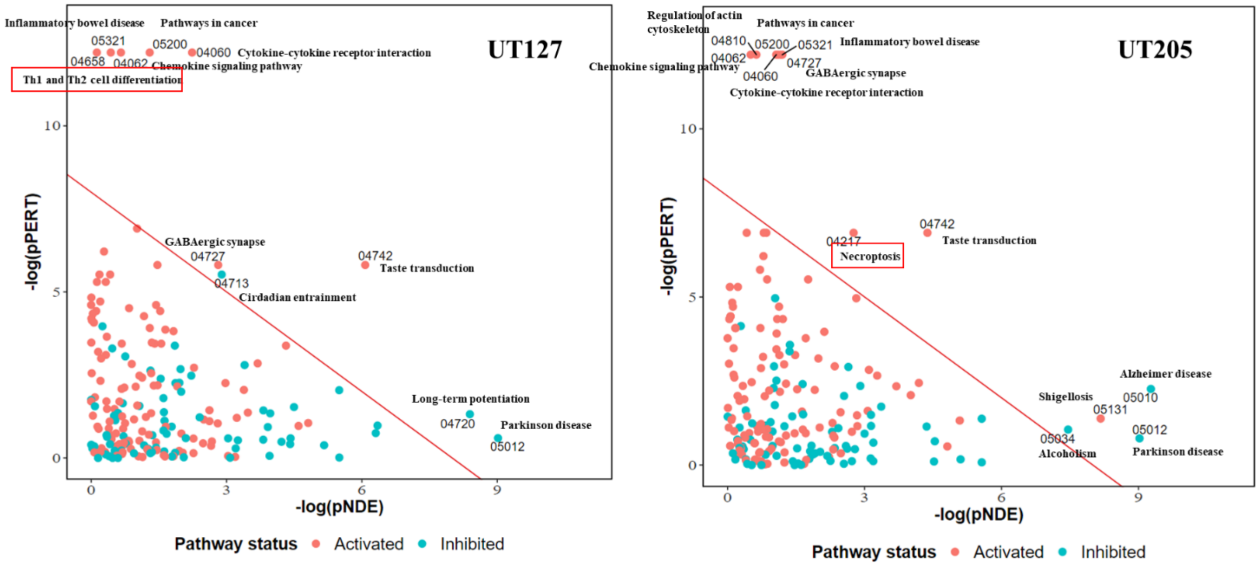

Figure S3. ***Signaling pathway impact analysis (SPIA)***. An impact analysis was used to investigate the perturbation in signaling pathways caused by the infection of hSMs with Mtb UT127 or UT205. A predominant enrichment of Th1 and Th2 cell differentiation functional category responded to UT127 infection while the necroptosis cell death functional category was over-represented in hSMs infected with UT205. The oblique red line represents the significance at 5% after the Bonferroni correction. $P_{(NDE)}$ measures the significance of a particular pathway as defined by an over-representation analysis of the number of DEGs observed on the pathway. pPERT reflects the amount of perturbation measured in each pathway and accounts for the topology or hierarchy of biological networks. The red and blue dots represent specific pathways. Red rectangles enclose significant pathways differentiating the macrophage's innate response to UT127 or UT205 infection.

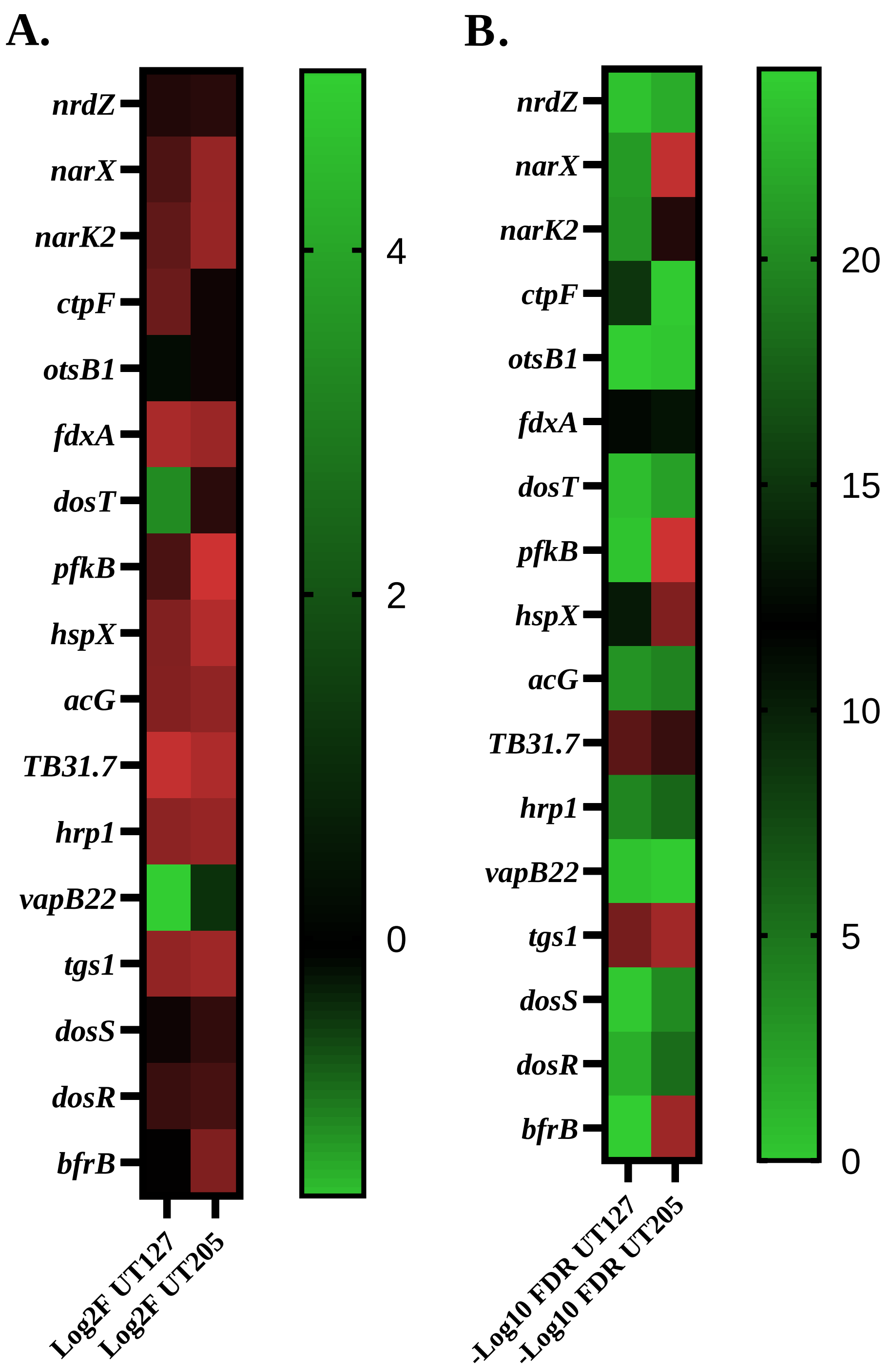

Figure S4. ***dosR* regulon expressed genes are preferentially expressed by Mtb UT205**. The heat maps display the expression (Log2 Fold, **A**) and significance (False Discovery Rate, FDR) of *dosR* regulon expressed genes by Mtb UT127 and UT205 within 6 hours of infection.

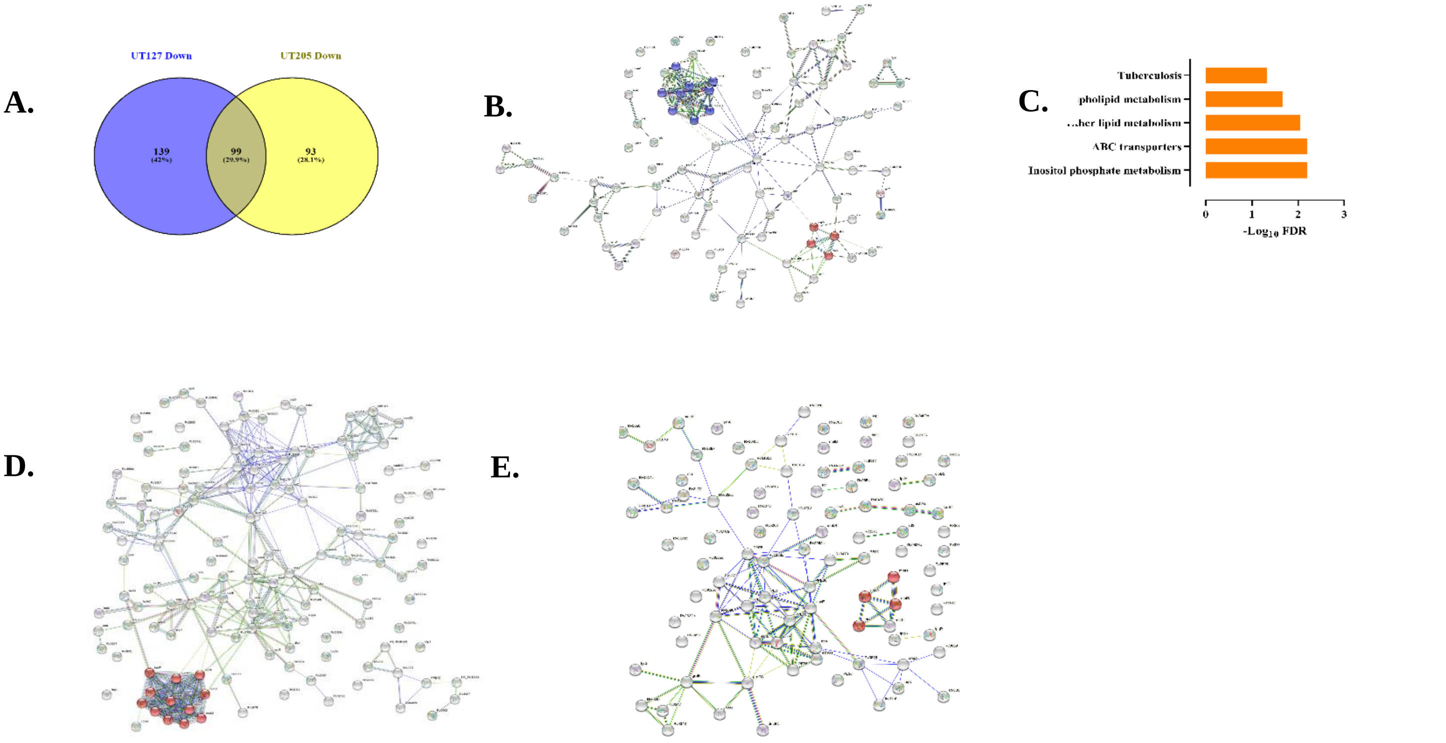

Figure S5. ***Protein-Protein interaction network of common and unique downregulated genes expressed by Mtb within 6 hours of hSMs infection***. (**A**) Protein-Protein interaction network of commonly downregulated differentially expressed genes (n=99) deduced from the String database. The 2 top GO categories, ABC transporters (FDR 0.0003) and inositol phosphate metabolism (FDR 0.0003), are encircled. Right: Bar chart showing the significant GO terms associated with the downregulated genes. (**B**) Significant KEGG pathways (FDR <0.05) associated with the 99 common differentially expressed genes by hSMs infected for 6 hours with *Mtb*. (**C**) Protein-Protein interaction network of unique differentially expressed downregulated genes (n=139) of *M. tuberculosis* UT127 within 6 hours of infection of hSMs. Encircled genes are associated with the GO term oxidative phosphorylation (FDR 1.85E-5).

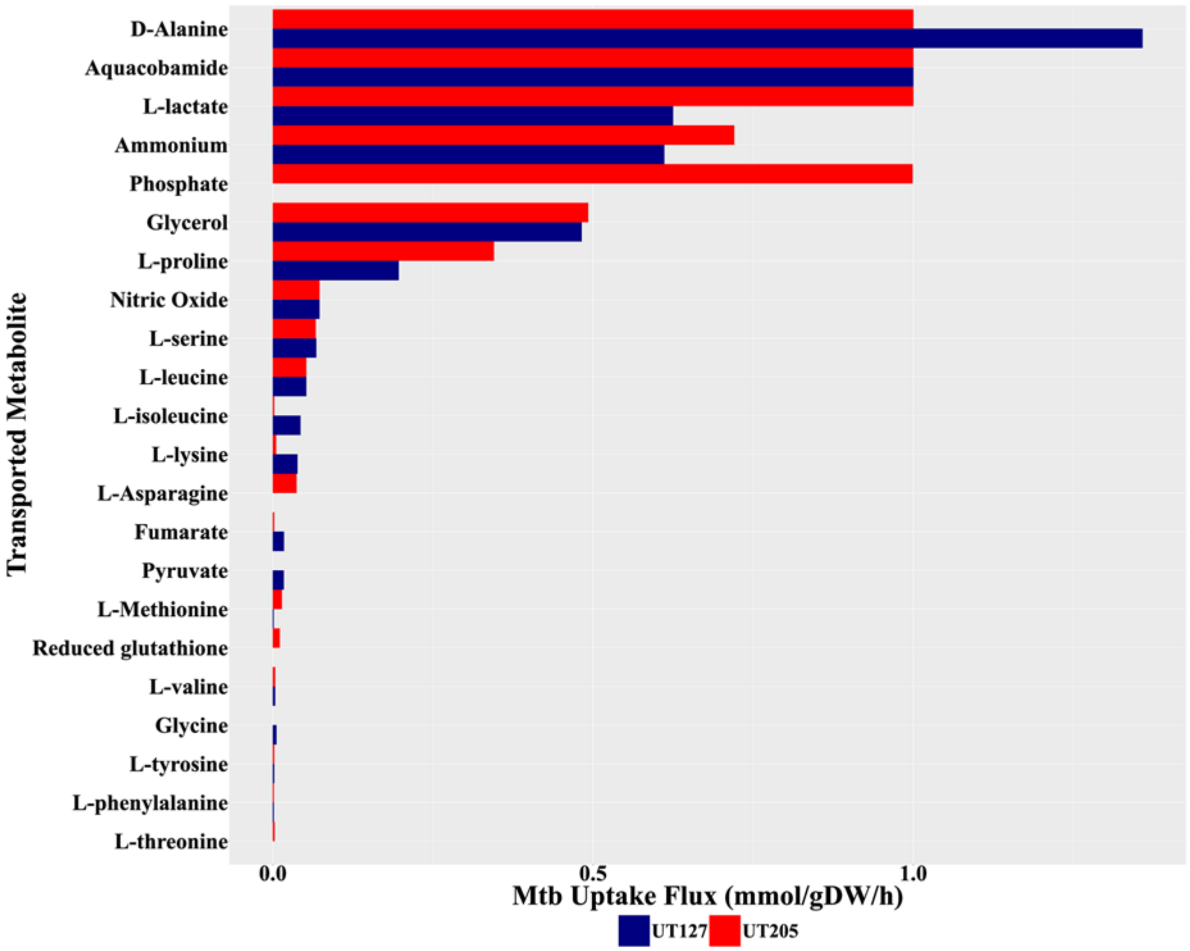

Figure S6. ***iMAT-based prediction of phagosome transported metabolites into Mtb***. The Integrative Metabolic Analysis Tool (iMAT) procedure was used to predict fluxes of metabolites exchanged between the hSMs phagosome and Mtb (UT127 and UT205). Flux distribution was computed by integrating the transcriptomics data into the Host-Pathogen genome-scale model. Here, up and down-regulated genes ($|\log 2FC| \geq 1.5$, $pvalue < 0.05$, $FDR < 0.05$) were used to constraint the optimization used in the iMAT procedure. No constraints were set for the biomass reactions.

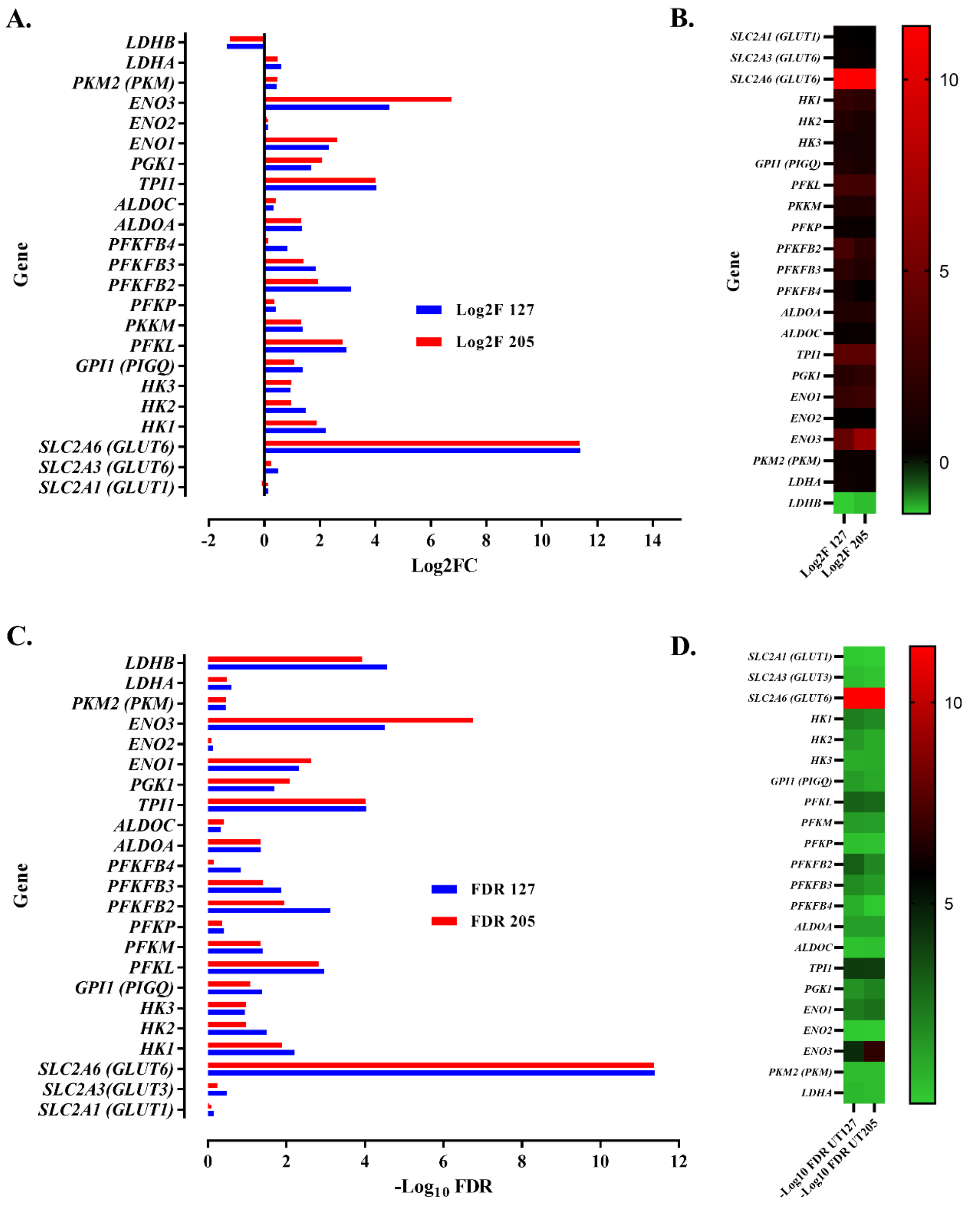

Figure S7. ***Expression of genes associated with glycolysis in hSMs infected with Mtb UT127 or UT205***. A comparison of the expression (Log2FC) (**A**) and the False Discovery Rate (Log10 FDR) (**C**) associated with macrophage (hSMs) genes participating in the glycolytic pathway in response to *M. tuberculosis* UT127 and UT205 infection is shown. (**B-D**) Heat map of the Log2FC (**B**) and the Log10 FDR (**D**) values of the glycolytic genes expressed by hSMs in response to the infection with UT127 and UT205. The upregulation of

genes participating in glucose transport, mainly *GLUT6*, the glycolytic enzymes *HK1* and *HK2*, the phosphofructokinase *PFKFB3*, the enolases *ENO3* and *ENO2*, and the lactate dehydrogenase *LDHA* is observed. The expression of the *PFKFB3*, *HIF1A*, *HK1*, *HK2*, *PDP1* and *PDP2* genes was higher in hSMs infected with UT127 compared with UT205, suggesting stronger glycolysis induced by the infection with UT127 compared with UT205.

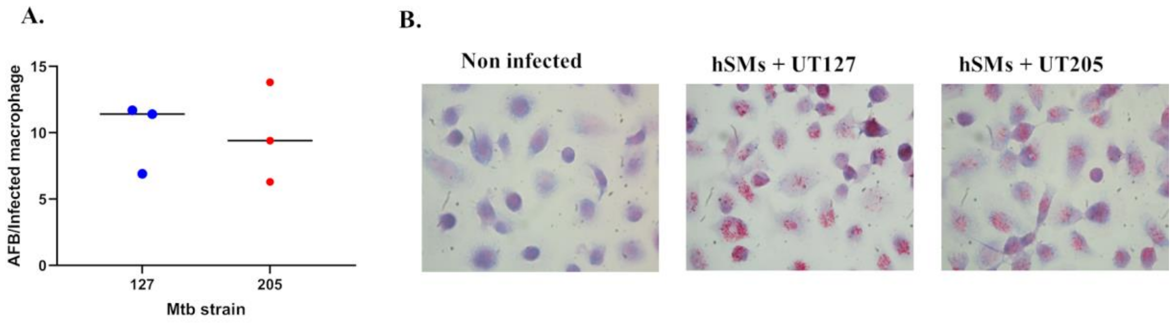

Number of Acid-Fast Bacilli (AFB) per infected macrophage in human splenic macrophages infected with M. tuberculosis UT127 and UT205. 3 x 10^5 human splenic macrophages (hSMs) were infected for 6 hours with an MOI of 10:1 of either M. tuberculosis (Mtb) UT127 or UT205. Briefly, macrophages were seeded in 24-well plates for 24 hours, washed with a warm (37 ºC) antibiotic-free medium to eliminate non-adherent cells, resuspended in RPMI-1640 supplemented with 10% AB+ medium in the absence of antibiotics. Then, approximately 3 x 10^6 CFUs of Mtb were added, and the plates were centrifuged 700 x g for 1 minute to increase the efficiency of Mtb: Macrophage contact and cultured for 6 additional hours. At the end of the phagocytosis period, the wells were extensively washed with warm (37 ºC) antibiotic-free PBS to eliminate extracellular bacteria. Cells were fixed with 0.25% glutaraldehyde for 10 minutes at 37 ºC and then air-dried. To estimate the % of infected macrophages and the AFB/infected macrophage, cells were stained by the Ziehl-Neelssen method. Initially, cells were stained with a pre-heated (50 ºC) Fucsin solution for 3 minutes, rinsed with tap water to eliminate the excess of stain, decolorated with acid-alcohol, then incubated with a solution of methylene blue for 20 seconds. To eliminate the excess of methylene blue, cells were washed with tap water and air-dried. To estimate the % of infected macrophages, at least 200 hundred cells were counted by selecting random fields (1000X) and counting cells with at least 1 AFB. To calculate the number of AFB/infected macrophages, the method described by [103] was used. The cells were classified as containing no AFB, 1-5 AFB, 6-10 AFB, 11-20 AFB, and >20 AFB, and the geometric mean calculated by 2 independent observers (**A**). Data were obtained from 3 independent experiments with the following results: UT127: 11.4, 6.9, and 11.7 AFB/infected macrophage; UT205: 9.4, 6.3, and 13.8 AFB/infected macrophage (Mann-Whitney P-value=0.9). In average, the % of infected macrophages was 88.6 % (range, 84.2-96.5, n=3) (**B**)
